# Beyond Antimicrobial Defense: Insect Antimicrobial Peptides as Neuroimmune Effectors and Insect-Derived Peptide Resources

**DOI:** 10.3390/insects17070694

**Published:** 2026-07-03

**Authors:** Jie He, Xinyu Li, Hongli Ji, Xi Chen, Yunjia Xiang

**Affiliations:** 1 Institute of Neurology, Sichuan Provincial People’s Hospital, School of Medicine, University of Electronic Science and Technology of China, Chengdu 610054, China; nicolas63@163.com (J.H.); 13308050816@163.com (X.L.); 2 Institute of Plant Protection, Sichuan Academy of Agricultural Sciences, Key Laboratory of Integrated Crop Pest Management in Southwest China, Ministry of Agriculture, Chengdu 610066, China; jihongli@scsaas.cn

**Keywords:** insect antimicrobial peptides, neuroimmunity, insect venom, peptide resources, translational scaffolds, nervous system

## Abstract

Insect antimicrobial peptides (AMPs) are classically viewed as terminal effectors of innate immunity, but emerging evidence suggests that some can also shape defined neural states. In this Review, we argue that insect systems provide a powerful framework for resolving immune–brain communication at the level of individual peptide effectors, because genetically tractable innate-immune pathways allow pathway activation to be distinguished from peptide-specific effector function. Rather than surveying AMP families exhaustively, we focus on representative cases in which peptide identity, source, and timing can be linked to sleep, memory-related plasticity, and responses to acute injury. These studies show that the neural consequences of AMP induction cannot be inferred from pathway activation alone, but require peptide-level analysis of effector identity, cellular context, and exposure logic. This perspective also raises the question of translational potential. At present, direct biomedical development of endogenous insect AMPs in neural contexts remains limited, whereas more tangible applied interest has centered on insect venom peptides that share AMP-like physicochemical features. We therefore discuss insect venoms separately from endogenous AMP physiology. Venom peptides are not physiological equivalents of endogenous insect AMPs, but represent evolutionarily diversified AMP-like templates for scaffold discovery, mechanistic probing, and therapeutic engineering. Together, this review develops a peptide-level perspective on insect neuroimmune biology while highlighting insect venoms as a valuable, but highly constrained, source of templates for biomedical discovery.

## Simple Summary

Insects provide useful model systems for studying how immune peptides influence the nervous system. Recent studies show that some insect antimicrobial peptides are not only immune effectors, but can also affect neural states such as sleep, memory-related plasticity, and responses to injury. Although endogenous insect antimicrobial peptides currently have limited direct biomedical application in neural contexts, insect venom peptides offer a diverse source of AMP-like bioactive templates. These venom-derived peptides are not physiological equivalents of endogenous AMPs, but they may provide useful scaffolds for mechanistic studies and future peptide engineering.

## 1. Introduction

Antimicrobial peptides (AMPs) are diverse, short innate immune effectors typically enriched in cationic and hydrophobic residues. These physicochemical traits render them amphipathic, facilitating their interaction with negatively charged microbial surfaces [1]. Functionally, however, AMPs do not operate through a single mechanism. Many AMPs kill microbes by disrupting membranes, whereas others target intracellular processes or regulate immune signaling [2,3]. This broader activity is important for understanding AMP function in host tissues, including the central nervous system (CNS).

In the CNS, AMPs may influence neural tissue through activities beyond antimicrobial defense, including effects on membrane dynamics, immune signaling, and cellular stress responses. These processes are closely linked to neuronal excitability, synaptic plasticity, barrier integrity, and circuit stability [3,4,5]. Therefore, AMP induction in neural contexts depends on which peptides are produced, where they originate, how long they persist, and how these exposure patterns shape neural outcomes.

Insect models, especially *Drosophila melanogaster*, are well suited for addressing these questions. Their genetic tractability allows individual AMP effectors to be tested directly, rather than inferring peptide function from broad Toll or Imd pathway activation alone. In addition, the fly brain is protected by a glial blood–brain barrier, which provides an experimentally accessible interface for distinguishing systemic immune responses, barrier-associated signaling, and local neural effects. Several studies have used this experimental strength to link specific AMP-family peptides to defined neural phenotypes. Nemuri promotes recovery sleep, Diptericin B (DptB) supports long-term memory formation, and Metchnikowin (Mtk) contributes to adverse outcomes after traumatic brain injury [6,7,8]. Recent syntheses further indicate that AMPs can intersect with neuronal function and disease-related neural outcomes, including sleep regulation, memory formation, traumatic brain injury, and neurodegenerative disease mechanisms [9]. These findings highlight insects as tractable systems for examining how endogenous peptide effectors influence defined neural states. This peptide-level framework also raises a broader translational question: whether insect-derived peptides can serve as molecular templates for neurobiological research and peptide engineering. Because direct biomedical development of endogenous insect AMPs in neural contexts remains limited, we next consider insect venoms as a distinct AMP-like peptide resource.

Insect venoms represent evolutionarily diversified peptide reservoirs that have been shaped for rapid bioactivity in heterologous target organisms [10,11,12]. Many venom peptides share AMP-like physicochemical features, including compact size, cationicity, amphipathicity, and membrane reactivity, as illustrated by melittin-like toxins, mastoparan-like peptides, and ant ponericins [11,13,14]. In venoms, however, these features are often coupled to activities directed toward excitable tissues, including ion-channel modulation and, in some cases, direct membrane activity. Thus, venom peptides should not be treated as physiological equivalents of endogenous insect AMPs. Rather, they are best viewed as bioactive templates for neurobiological probe development and, more selectively, peptide engineering. Their potential value depends not only on pharmacological potency, but also on whether useful activity can be separated from nonspecific toxicity and evaluated against selectivity, safety, delivery feasibility, and developability [11,15,16].

Although insect AMPs have been reviewed extensively, most existing ones focus on AMP diversity, regulatory pathways and antimicrobial activity [1,17,18]. Less attention has been given to how individual insect AMPs influence defined neural states. Our review addresses this gap by focusing on representative examples linked to sleep, memory-related plasticity, and acute injury. We also organize these examples around peptide identity, tissue source, timing, and exposure duration. We then discuss insect venoms as a distinct source of AMP-like peptide templates, emphasizing both their value for probe development and engineering and the limitations that constrain translational interpretation. Together, these perspectives support a peptide-level approach to insect neuroimmune biology and provide a cautious basis for evaluating insect-derived peptide scaffolds in biomedical discovery.

## 2. Canonical AMP Control Logic and Why Insects Are Well Suited to Effector-Level CNS Dissection

**Figure 1:**
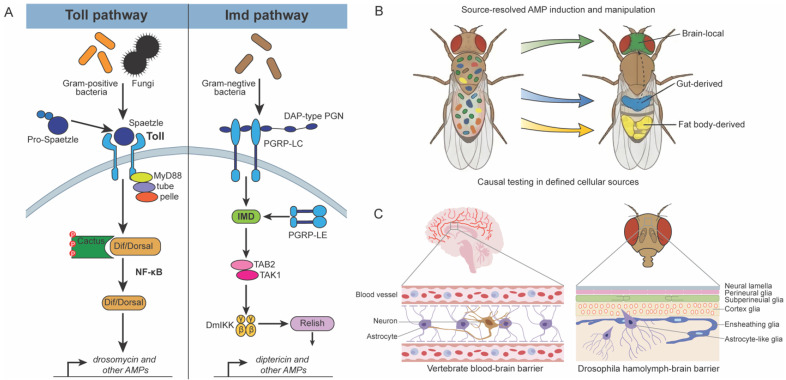
Canonical Toll/Imd AMP control logic and source-resolved neuroimmune features enabling effector-level central nervous system (CNS) dissection in *Drosophila*. (**A**) Simplified schematic of the canonical Toll and immune deficiency (Imd) pathways that regulate antimicrobial peptide (AMP) induction in *Drosophila*. Fungal and Gram-positive cues activate Spaetzle–Toll signaling, relieving Cactus-mediated inhibition and enabling Dif/Dorsal-dependent transcription of antifungal AMPs such as Drosomycin. Gram-negative bacterial diaminopimelic acid (DAP)-type peptidoglycan activates PGRP-LC/PGRP-LE–immune deficiency (Imd) signaling, culminating in Relish-dependent transcription of antibacterial AMPs such as Diptericin. (**B**) Source-resolved immune effector programs in *Drosophila* can be distinguished across the brain, gut, and fat body, enabling tissue-specific analysis and manipulation of AMP-related signaling. The added gut-to-brain-surface cue (dashed line) highlights that gut-derived microbial signals can engage barrier-associated neuroimmune signaling at the brain surface, including perineurial glia. (**C**) Barrier-organized neuroimmune interfaces in vertebrates and flies. The vertebrate blood–brain barrier is centered on the vascular interface associated with neurons and astrocytes, whereas the *Drosophila* hemolymph–brain barrier is formed primarily by glial layers, including the neural lamella, perineurial glia, and subperineurial glia, with additional interactions from cortex glia, ensheathing glia, and astrocyte-like glia.

Insect AMP induction is a genetically organized transcriptional program governed primarily by the canonical Toll and immune deficiency (Imd) pathways (Figure 1A). The Toll pathway is classically engaged during antifungal and Gram-positive bacterial responses through the extracellular proteolytic activation of Spaetzle. This activation initiates a signaling cascade that relieves Cactus-mediated inhibition and allows the nuclear factor kappa B (NF-κB) family factors Dif and Dorsal to drive antifungal AMP expression, including drosomycin [19,20,21]. By contrast, the Imd pathway is activated mainly by diaminopimelic acid (DAP)-type peptidoglycan sensed by peptidoglycan recognition protein (PGRP)-LC and PGRP-LE, which signals to promote Relish-dependent antibacterial AMP induction [22,23,24,25]. Importantly, this regulatory architecture is combinatorial rather than rigidly binary. Individual AMP genes respond differently to pathogen class, tissue context, and transcription-factor input, allowing pathway activation to generate context-specific peptide outputs rather than a uniform AMP response [19,21,26].

The non-equivalence between upstream pathway activation and specific peptide output creates an important interpretive challenge for neuroimmune analysis. In insects, AMP production can arise from spatially distinct anatomical sources (Figure 1B). Systemic infection classically induces robust AMP secretion from the fat body into the circulating hemolymph, whereas more localized expression can occur in peripheral epithelia and at brain- or barrier-associated sites [26,27]. As a result, the nervous system may encounter AMP-related signals through hemolymph-borne exposure, barrier-associated signaling, or local induction near neural tissue.

The *Drosophila* model is particularly advantageous because these spatial variables can be addressed with both anatomical and genetic resolution. The fly CNS is enclosed by a glial blood–brain barrier (BBB), which separates neural tissue from the hemolymph and provides a defined interface for immune–brain communication [28,29,30,31] (Figure 1C). Importantly, this barrier is not merely a passive partition. Gut-derived microbial cues, for example, can engage NF-κB signaling in perineurial glia at the brain surface [32]. Moreover, cell-type-specific genetic tools in *Drosophila* can help determine whether a neural phenotype reflects broad immune activation or whether it is driven by a particular AMP in a defined tissue context [7,27,32].

## 3. Endogenous Insect AMPs in Neural States: Peptide-Specific Evidence, Source, and Timing–Duration Patterns

**Figure 2:**
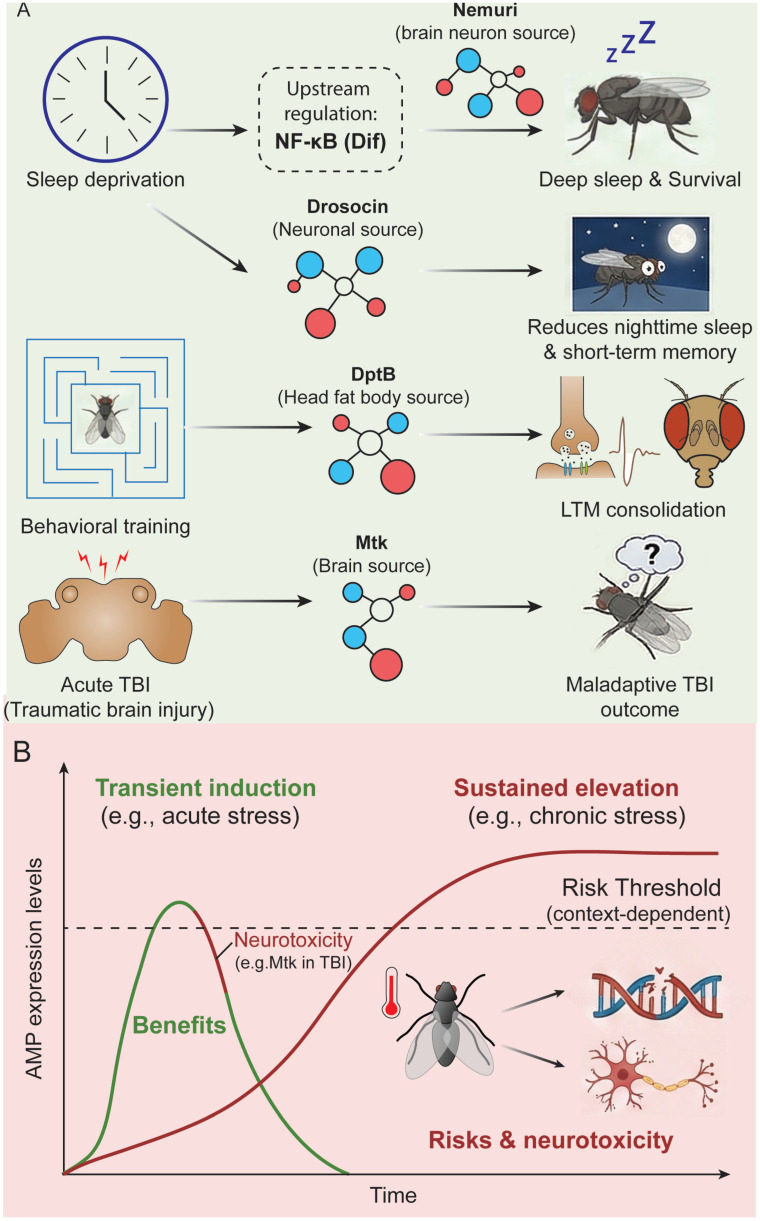
Peptide-resolved evidence linking endogenous insect antimicrobial peptides to neural states. (**A**) Representative *Drosophila* examples. Sleep deprivation engages immune-to-sleep regulation involving the NF-κB factor *Dif* and induces neuronal Nemuri, which promotes deep and recovery-associated sleep. *Drosocin* is shown as a candidate example because sleep-deprivation-associated and targeted-expression data suggest that neuronal Drosocin reduces nighttime sleep and short-term memory, although endogenous loss-of-function evidence remains limited. Behavioral training recruits head-fat-body-derived DptB for long-term memory consolidation, showing that extra-neural peptide sources can influence central neural states. Acute traumatic brain injury induces brain Metchnikowin (Mtk), and loss of *Mtk* reduces mortality and behavioral impairment, linking this peptide to maladaptive injury responses. Together, these examples emphasize that AMP-associated neural effects vary with peptide identity, anatomical source, induction context, and evidence strength. (**B**) Timing-duration framework for AMP-associated neural outcomes. Transient, state-linked induction, as illustrated by Nemuri and DptB, is often associated with adaptive state regulation. Sustained, ectopic, or poorly restrained AMP-associated activity, including the Pirk-AttacinD model discussed in the text, is more often associated with neural dysfunction or disease-related phenotypes. This relationship is not absolute, because Mtk illustrates that acute induction can also be maladaptive in a specific injury context. The model is intended as a context-dependent interpretive framework, not a universal protective-or-harmful rule. All elements are schematic and not to scale.

Building on this experimental tractability, current evidence suggests that selected endogenous insect AMPs can influence defined neural states in vivo. These studies are most informative when the peptide effector, anatomical source, upstream induction pathway, timing and duration of exposure, and physiological or pathological context can be resolved experimentally. This organization helps distinguish peptide-specific effects from broader consequences of immune-pathway activation [6,7,8] (Figure 2).

One clear example comes from sleep homeostasis in *Drosophila*. Sleep deprivation induces the antimicrobial peptide Nemuri (encoded by *nemuri*) in a small subset of brain neurons, where it promotes deep sleep and contributes to rebound sleep [6]. Recent work further links this peptide-level response to upstream immune regulation: the NF-κB factor Dif is required for full *nemuri* induction and for behavioral recovery sleep after prolonged wakefulness, indicating that Nemuri acts as a defined peptide effector linking immune signaling to sleep regulation [33] (Figure 2A).

Importantly, sleep-associated AMP-family effects are not uniform. Sleep deprivation has been reported to increase Drosocin expression in neuronal contexts. Functional manipulation further suggests that the site of expression matters: neuronal expression of Drosocin reduces nighttime sleep and impairs short-term memory, whereas glial expression does not produce the same effect. This finding suggests that AMP-family peptides may influence neural phenotypes in a cell-source-dependent manner [34] (Figure 2A). However, because this evidence is based mainly on stress-associated expression and targeted expression assays rather than endogenous loss-of-function analysis, Drosocin should be interpreted as a candidate example rather than as definitive evidence for physiological AMP function.

A complementary example concerns memory-related plasticity. Following behavioral training, DptB is required for long-term memory consolidation but is dispensable for short-term performance [7] (Figure 2A). The key mechanistic point is that this requirement maps to the head fat body, a non-neuronal tissue surrounding the brain, rather than to a broadly neuronal AMP program. Thus, the *DptB* model separates peptide identity from tissue source and indicates that extra-neural immune peptides can influence central neural states in a training-dependent manner. However, the downstream CNS targets and receptor-level mechanisms through which head-fat-body-derived DptB influences memory consolidation remain incompletely defined. While other AMP-family candidates have also been implicated in neuronal phenotypes, the supporting evidence often remains fragmentary, relying primarily on stress-associated expression changes or gain-of-function assays rather than source-defined endogenous requirements [34].

A more recent disease-oriented example showed that loss of Pirk, a negative regulator of Imd/NF-κB signaling, produces age-dependent neurological abnormalities in *Drosophila*, including altered sleep patterns, impaired locomotion, and brain lesions. Importantly, these phenotypes are partially rescued either by knockout of the AMP gene *AttacinD* or by axenic rearing, indicating that both chronic AMP-associated immune output and gut microbial state contribute to the neural consequences of impaired immune restraint [35]. This model provides an important counterpoint to transient, state-linked AMP responses such as Nemuri. Nemuri represents a transient, state-linked AMP response that supports recovery sleep, whereas the Pirk–AttacinD model shows how poorly restrained AMP-associated immune activity can contribute to age-dependent neural dysfunction. Thus, AMP-associated immune outputs should be interpreted not simply by their induction, but by the regulatory and exposure context in which they occur.

Duration is one key factor that helps explain these divergent outcomes (Figure 2B). Transient induction, as illustrated by Nemuri during sleep recovery and DptB after behavioral training, is more consistent with adaptive state regulation. By contrast, sustained or ectopic AMP-associated immune activation is more often linked to neural dysfunction, locomotor decline, reduced lifespan, or neuropathology [6,7,27,36]. This distinction is not absolute, however. In acute traumatic brain injury, Mtk is induced in the brain, yet loss of *Mtk* reduces early mortality and behavioral impairment. Thus, Mtk provides an example in which injury-associated AMP induction is linked to maladaptive outcomes in a specific acute trauma context [8] (Figure 2). Duration is, therefore, best viewed as a risk-shaping variable rather than a simple determinant of benefit or harm.

Overall, in vivo studies of endogenous insect AMPs support a context-dependent model of peptide-resolved neuroimmune regulation. Rather than acting as uniformly protective or harmful effectors, these peptides appear to influence neural outcomes according to peptide identity, anatomical source, upstream immune regulation, exposure duration, gut microbial state, and disease context. Recent neurodegeneration-focused syntheses support this disease-oriented perspective by discussing links between persistent or ectopic AMP expression and pathology across *Drosophila* models and beyond [37]. In parallel, primary evidence indicates that AMP overexpression can accelerate aging through cytotoxic effects in *Drosophila* tissues [38]. However, this pathogenic paradigm requires careful contextualization. Systematic deletion studies reveal that endogenous AMPs do not universally drive aging pathology; rather, they often preserve host fitness by restricting age-related dysbiosis [39]. Consequently, endogenous insect AMPs are best conceptualized as genetically tractable, disease-relevant effectors rather than direct therapeutic candidates. Ultimately, the mere presence of an AMP is insufficient to predict neural function; the defining factors dictating biological outcomes are precisely when, where, and for how long a specific peptide operates.

## 4. Constraining AMP-like Liability: Membrane Shielding, Controlled Maturation, and Spatial Segregation

A central question raised by endogenous AMP biology is whether membrane-active peptide effectors carry an intrinsic liability for host-tissue toxicity, including potential risk in neural contexts. This concern is biologically plausible, because many host-derived AMPs are short, cationic, and often membrane-active, and insect venom peptides use related physicochemical properties to act on excitable tissues and, in some cases, host membranes with high potency [40,41]. The key issue, therefore, is how insects restrict and control this membrane-active potential, especially in vulnerable tissues such as the nervous system. More broadly, this concern fits with the view that host-defense peptides can exert diverse biological effects that depend on target accessibility and tissue environment [42].

The clearest direct evidence for endogenous protection against AMP-mediated self-toxicity comes from the *Drosophila* Turandot proteins. These stress-induced host factors protect tissues from host AMP cytotoxicity by binding to host membranes and masking negatively charged phospholipids that would otherwise remain susceptible to cationic peptide attack [43]. This mechanism is conceptually significant because it identifies the host membrane surface itself as a regulated determinant of peptide injury. More generally, the damaging potential of cationic antimicrobial peptides depends strongly on membrane composition and target accessibility, particularly the availability of anionic phospholipids that favor peptide binding and insertion [44]. Accordingly, one way insects can limit AMP self-toxicity is not by eliminating peptide activity, but by limiting access to vulnerable host membrane surfaces. Although the Turandot mechanism was demonstrated in the tracheal system rather than in neural tissue [43], it provides a useful conceptual precedent for neuroimmune biology by showing that host membrane accessibility can be actively regulated to limit AMP-mediated self-damage.

A second, more indirect but still informative layer of control is that insect AMPs are commonly produced through regulated maturation and spatially restricted deployment. Classical work showed that insect defensin is synthesized as a prepro-peptide, and more recent studies indicate that some *Drosophila* AMP families are produced as larger precursor proteins that require proteolytic processing to generate mature peptides, as exemplified by Baramicin A [45,46]. In parallel, many inducible *Drosophila* AMPs are produced predominantly by the fat body and secreted into the hemolymph rather than constitutively expressed throughout neural tissue [47]. Although these observations do not by themselves demonstrate a dedicated anti-neurotoxicity program, they support the idea that peptide toxicity may be constrained by controlled maturation and spatial segregation, thereby providing plausible ways to reduce indiscriminate exposure of host tissues to mature effectors.

Such upstream control mechanisms become particularly important when immune activation is sustained rather than transient. In chronic inflammatory settings, risk is likely to arise not only from increased effector abundance, but also from loss of the spatial and temporal restraint that normally limits AMP-like activity. Consistent with this view, glial ATM deficiency in *Drosophila* is associated with chronic activation of innate immune response genes together with progressive neurodegeneration [48]. Similarly, loss of Dnr1-mediated restraint results in sustained Imd-pathway activation, elevated AMP expression, and age-dependent neuropathology; importantly, the same study further showed that neural overexpression of individual AMP genes is itself sufficient to induce neurodegeneration [36]. Read in this context, these models point to a more specific pathogenic principle: persistent immune induction can become deleterious when it outlasts or overrides the mechanisms that normally restrict AMP-like activity in space and time.

This framework also has translational relevance. Endogenous insect AMP biology shows that peptide activity and toxicity are closely linked, and that beneficial effector functions require mechanisms that limit inappropriate host exposure. However, direct biomedical development of endogenous insect AMPs in neural contexts remains limited. This limitation provides a rationale for considering insect venoms as a distinct AMP-like peptide resource, where similar physicochemical features have been diversified into broader bioactivities that may be explored for scaffold discovery and engineering [16].

## 5. From Endogenous Effectors to Engineering Space: Insect Venoms as AMP-like Peptide Template Reservoirs

Insect venoms represent a distinct expansion of the insect peptide landscape. They extend the discussion from endogenous immune effectors to a broader reservoir of bioactive peptide templates shaped by ecological and evolutionary selection. Here, venom peptides are considered as insect-derived resources for scaffold discovery and engineering, while remaining conceptually separate from endogenous AMP physiology [10].

Many venom peptides retain AMP-like features, including compact size, cationicity, amphipathicity, and membrane activity, but in venoms these properties have been tuned for potent action on excitable tissues rather than for controlled physiological deployment [11,14]. The transition from endogenous AMPs to venom peptides is therefore best understood as a shift from physiological effectors to evolutionarily enriched peptide repertoires with greater value for probe development and scaffold engineering [40]. That expanded utility also brings greater liability, because the same membrane-active properties that confer potency can narrow the therapeutic window and increase target-independent toxicity. Insect venoms are thus best viewed as structured reservoirs of candidate peptide templates whose translational relevance depends on target selectivity, safety margins, delivery properties, and overall developability rather than on potency alone [10,11,14].

One better-supported use of venom peptides is as mechanistic probes or scaffolds for defined target engagement. The bee venom component apamin, for example, is a compact, disulfide-stabilized blocker of small-conductance Ca^2+^-activated K^+^ (SK/K_Ca_2) channels, which regulate neuronal firing precision and excitability [49,50]. Consistent with this mechanism, SK blockade can alleviate behavioral deficits in dopamine-depleted rats [51]. Likewise, ant venom peptides, including poneratoxin and recently characterized voltage-gated sodium channel (NaV)-targeting ant toxins, serve as probes of NaV channel gating, helping define thresholds of neuronal excitability, nociceptor activation, and pain-linked sensitization [52,53,54]. One important value of such peptides is that they help clarify the structural motifs and target interactions required for selective engagement. In this respect, they function most clearly as component-resolved mechanistic scaffolds rather than as direct therapeutic agents [15,16,40].

A distinct and more challenging class comprises membrane-active peptides and multicomponent venom systems, for which the central engineering problem is to separate useful signaling from intrinsic toxicity. Honeybee venom illustrates this issue well. In mammalian Parkinson’s disease (PD) models, repeated administration of whole bee venom has been reported to protect nigrostriatal dopaminergic neurons, whereas apamin reproduces only part of this effect, indicating that the reported neuroprotective activity of bee venom cannot be assigned to a single component without further deconvolution [55]. Component-level studies place different bee venom constituents at different translational positions. Fractionation studies have reported robust neuroprotective activity for bee venom phospholipase A2 (bvPLA2), whereas melittin has long been regarded primarily as a liability because of its cytolytic activity [56]. Disease-oriented studies further support the need for component-level interpretation. In Alzheimer’s disease-related models, bee venom phospholipase A2 has been reported to reduce amyloidogenesis and neuroinflammation through inhibition of STAT3 signaling in Tg2576 mice [57]. This finding extends the relevance of bvPLA2 beyond Parkinson’s disease models, but it also reinforces the need to separate component-specific immunomodulatory effects from the broader pharmacology and toxicity of whole bee venom. More recent work suggests that, under carefully titrated sub-toxic conditions, melittin can also engage neuroprotective signaling programs, including tropomyosin receptor kinase B (TrkB)/cyclic AMP response element-binding protein (CREB)/brain-derived neurotrophic factor (BDNF)-associated pathways, in neuronal stress models [58]. Wasp venom-derived peptides provide additional examples of venom components with neurological disease-related activity. Mastoparan-M and vespakinins have been reported to reduce neuronal death, oxidative stress, inflammation, apoptosis, and BBB damage following ischemic stress [59,60]. More recent work further suggests that Mastoparan M promotes functional recovery after stroke by activating autophagy and inhibiting ferroptosis [61]. These findings broaden the neurological disease relevance of wasp venom-derived peptides, but they should still be interpreted as preclinical and mechanism-generating evidence rather than as direct therapeutic validation. Within a mechanism-guided framework, these findings are best treated as hypothesis-generating rather than as direct evidence of clinical potential, because component-resolved mechanisms, long-term safety margins, delivery feasibility, membrane toxicity, and developability remain unresolved. Together, these studies suggest that useful bioactivity and toxic liability may be partially separable under defined experimental conditions. The available clinical evidence should be interpreted more cautiously. Although a randomized, double-blind trial reported that monthly whole-bee-venom injections were safe in non-allergic subjects, this finding provides only limited safety context for the whole venom preparation and does not establish the therapeutic window of individual venom components. Rigorous component-resolved optimization and dose–toxicity evaluation remain necessary before broader therapeutic claims can be made [62].

A third biomedical application of venom-derived scaffolds lies in delivery engineering. This application is particularly relevant to neurotherapeutic development because many peptide-based candidates are limited not only by target potency or selectivity, but also by poor access to the vertebrate CNS. Several venom-derived motifs have therefore been repurposed as carriers or shuttle elements for CNS delivery. Transportan, a chimera of galanin and the wasp peptide mastoparan, is a widely used cell-penetrating peptide (CPP) [63]. More recent work has generated BBB-shuttle variants, including minimized apamin-derived motifs such as MiniAp-4, with the aim of preserving transport properties while reducing target-related toxicity and immunogenic risk [64]. These delivery-oriented examples reinforce a broader translational principle. Insect venoms are most valuable not simply as sources of potent bioactive peptides, but as reservoirs of structural and functional motifs that can be redesigned for safer and more selective applications. Their development potential should be evaluated by whether optimized scaffolds retain the desired activity, separate useful function from broad lytic, hemolytic, or off-target effects, enable realistic CNS delivery, and remain manufacturable within an acceptable safety window [65]. In neurodegenerative and neurological disease contexts, venom-derived insect peptides are therefore best viewed as candidate scaffolds or mechanistic probes for component-resolved optimization rather than as ready therapeutic agents.

## 6. Conclusions

**Figure 3:**
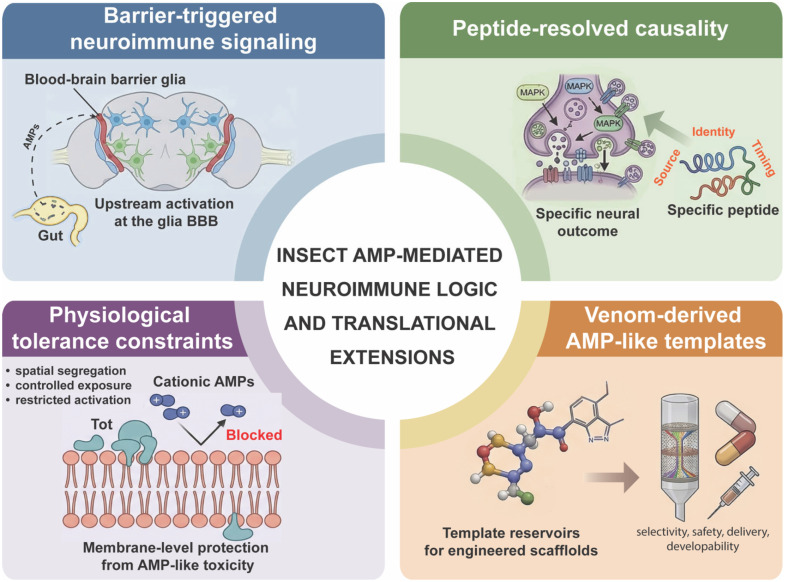
Conceptual framework for peptide-resolved insect neuroimmune biology and cautious evaluation of venom-derived peptide templates. Barrier-triggered neuroimmune signaling at the insect blood–brain barrier. Barrier-associated glia can integrate peripheral and microbial cues and initiate immune signaling at the brain surface. Peptide-resolved causality. Insect genetic tools allow individual peptide effectors to be separated from broad Toll/Imd pathway activation and tested in defined neural phenotypes. Interpretation should consider peptide identity, source, timing, duration, and evidence strength. Physiological constraints on AMP-like toxicity. Endogenous AMP-like activity may be limited by spatial segregation, controlled maturation or exposure, and membrane-level protection, which together can reduce inappropriate host-tissue damage. These are presented as restraint principles rather than proof of a dedicated neural protection program. Venom-derived AMP-like templates as a distinct translational resource. Insect venom peptides are not physiological equivalents of endogenous AMPs. They expand the design space for mechanistic probes, delivery motifs, and engineered scaffolds, but their value depends on component-level deconvolution and filtering for selectivity, safety margin, delivery feasibility, toxicity, manufacturability, and overall developability rather than potency alone.

Insect neuroimmunology broadens the study of host-defense peptides by showing that endogenous AMPs can act beyond terminal antimicrobial defense and may influence neural states in a context-dependent manner. Available evidence indicates that these effects depend on peptide identity, anatomical source, timing, exposure duration, and physiological or pathological context. At the same time, AMP-like activity carries intrinsic liabilities, especially when membrane-active peptides are produced inappropriately or persistently, making spatial restriction, temporal control, and membrane-level protection important considerations for interpreting AMP function in neural tissues. Extending this discussion to insect venoms identifies a related but distinct peptide resource. Venom-derived peptides can provide useful scaffolds for neuropharmacological probing, delivery engineering, and candidate peptide design, but their value cannot be inferred from potency alone. Progress will require component-level deconvolution and mechanism-guided engineering to separate useful target engagement or delivery motifs from broad lytic, hemolytic, or off-target toxicity. Overall, these lines of evidence support a peptide-level approach to insect neuroimmune biology and provide a cautious basis for evaluating insect-derived peptide scaffolds in biomedical discovery and engineering (Figure 3).

## Data Availability

No new data were created or analyzed in this study. Data sharing is not applicable to this article.
